# Effect of the health action process approach theory in patients undergoing total knee arthroplasty

**DOI:** 10.1038/s41598-025-34635-7

**Published:** 2026-01-05

**Authors:** Fangling Zhou, Yaruo Huang, Ruiqin Sha, Rui Hu, Xueyan Guan, Zexi Huang, Ying Tian, Nianqi Cui

**Affiliations:** 1 School of Nursing, Kunming Medical University, Kunming, China; 2 Department of Nursing, The First Affiliated Hospital of Kunming Medical University, Kunming, China; 3 Department of Nursing, The Third Affiliated Hospital of Kunming Medical University, Kunming, China

**Keywords:** Arthroplasty, replacement, knee, Rehabilitation, Exercise therapy, Evidence-based nursing, Self efficacy, Diseases, Health care, Health occupations, Medical research, Rheumatology

## Abstract

Rehabilitation training after total knee arthroplasty (TKA) is crucial for restoring joint function. This quasi-experimental enrolled 82 orthopedic patients from a Grade A tertiary hospital in Yunnan Province, who were divided into a control group (41 cases) and an experimental group (41 cases). Both groups received routine nursing care, while the experimental group additionally received a 3-month rehabilitation program based on the Health Action Process Approach (HAPA) theory combined with the best evidence for TKA rehabilitation. Preoperative indicators showed no significant difference in preoperative indicators between the two groups. Postoperatively, the experimental group demonstrated higher Hospital of Special Surgery (HSS) scores and Self-Efficacy for Rehabilitation Outcome Scale (SER) scores (P-adjusted = 0.020;P-adjusted = 0.006), as well as lower Numerical Rating Scale (NRS) scores and Western Ontario and McMaster Universities Osteoarthritis Index (WOMAC) scores (P-adjusted = 0.009, P-adjusted = 0.016). The research found that a rehabilitation program based on the HAPA theory is both scientific and effective, which can significantly relieve pain, improve joint function, and enhance patients’ self-efficacy in rehabilitation.

**Supplementary Information:**

The online version contains supplementary material available at 10.1038/s41598-025-34635-7.

## Introduction

Knee osteoarthritis (KOA) is typically a slow progressive disorder^1^. Its main clinical manifestations include joint pain, usually accompanied by swelling, deformity, and varying degrees of dysfunction, which limit patients’ mobility and represent one of the most common causes of disability among middle-aged and elderly individuals^2^. It has been reported that the global prevalence of KOA among people over 40 years old was 22.9% in 2020, and its incidence is expected to increase by 74.9% by 2050^3,4^. The high incidence rate, high disability rate, difficulty in achieving complete cure, and easy recurrence significantly affect the quality of life of patients and impose a heavy economic burden on individuals, families, and society^5^.

Total knee arthroplasty (TKA) is a preferred clinical treatment method for improving knee joint function and is commonly used as a final option for managing knee osteoarthritis^6,7^. With the growing prevalence of osteoarthritis, the number of TKA^8^ performed has also increased. Although significant progress has been made in TKA, patients may still experience limited range of motion, persistent pain, and restricted functional recovery following surgery^9^. However, most patients undergoing TKA expect to return to their normal life and work after surgery, including improvements in pain, function and quality of life^10^. This discrepancy between high rehabilitation expectations and potential adverse outcomes highlights the importance of rehabilitation. Therefore, postoperative rehabilitation exercises are essential for restoring joint function and have a significant influence on therapeutic outcome^11^.

Although numerous studies have investigated rehabilitation exercises after TKA, many lack strong theoretical grounding^12–14^, which may limit their effectiveness. Meanwhile, multiple theories and models exist for behavioral change, many neglect the context in which the target behavior occurs, devote limited attention to the reflective process, exhibit a static structure, and fail to clarify how behavior change upholds^15^. To address these limitations, the Health Action Process Approach (HAPA) was developed. HAPA emphasizes self-efficacy as a key construct for predicting and changing health behaviors^16^ and includes two stages: the motivational stage and the volitional stage^17,18^. The motivational stage is where individuals form their intentions, influenced by their risk perception, outcome expectations, and action self-efficacy. The volitional stage is critical for translating intentions into sustained actions through detailed action planning, proactive coping strategies, and the maintenance of self-efficacy when facing challenges. HAPA has demonstrated substantial positive effects in clinical practice and patients’ rehabilitation exercises^19–21^. Based on this framework, we developed a rehabilitation exercise intervention program based on the HAPA theory.

Currently, no universally accepted rehabilitation protocol exists for TKA patients, and rehabilitation paradigms are often institution or surgeon specific^22^.Our team has summarized the best available evidence on exercise interventions for patients with TKA^23^. In this study, we aim to integrate this evidence with HAPA to construct a postoperative rehabilitation exercise program and provide exercise guidance to hospitalized TKA patients. We also aim to evaluate the clinical effectiveness of this program, enhance postoperative rehabilitation efficiency, reduce joint dysfunction, and improve the patient’s’ quality of life.

Methods.

### Study design

This study employed a non-concurrent quasi-experimental design. The choice of this design was based on the average length of hospital stay for patients, which is approximately 14 days. To avoid cross-contamination in the same ward, patients scheduled to undergo TKA in different periods within the same department of the same hospital were selected as the control group and the experimental group, respectively. Therefore, we adopted a quasi-experimental approach rather than a fully randomized controlled trial.

### Participants

The study subjects were patients undergoing primary TKA in the Department of Orthopedics at a Grade A tertiary hospital in Yunnan Province. Patients scheduled to undergo TKA from May 2022 to August 2022 were enrolled in the control group, while those scheduled to undergo TKA from October 2022 to December 2022 were selected as the intervention group.

Inclusion criteria: (1) Participants must satisfy the diagnostic criteria for KOA as outlined in the Osteoarthritis Diagnostic and Treatment Guidelines of the Orthopedic Association of the Chinese Medical Association^24^; (2) Participants must be undergoing TKA for the first time in the affected joint; (3) Participants must have no severe dysfunctions of critical organs such as the heart, lungs, or kidneys;

Exclusion criteria: (1) Individuals who have undergone knee revision surgery, had malignant tumors, or had other related diseases; (2) Individuals with conditions that impair mobility, such as myasthenia gravis; (3) Individuals with mental illnesses or communication disorders. (4) unwilling participant to keep in the study.

### Sample size

The sample size was calculated using the formula for comparing means between two independent samples: n_1_ = n_2_ = 2×{(Z_α/2_+Z_*β*_)×σ/δ}^2^.

Here, σ represents the estimated value of the standard deviation of the two aggregates, which is generally assumed to be equal or taken as the square root of the combined variance, δ represents is the difference between the two means, and Z_α/2_ and Z_*β*_ are the values corresponding to the test level α and the probability of Type II error β, respectively, which were taken as α = 0.05 and β = 0.1, and the table Z_α/2_ = 1.96 and Z_*β*_ = 1.282 was checked. According to references^25,26^, the range of knee motion was used to calculate the index, take σ = 8.9, δ = 7.5, and substitute into the formula to get n1 = n2 = 30, considering the 25% loss of visit rate, at least 75 patients were collected in this study, and finally a total of 82 patients were collected.

### Blinding

In this study, it is not feasible to implement blinding for the medical staff and subjects involved in the intervention. However, blinding was applied to the professionals engaged in data collation and analysis to minimize bias.

### Intervention

A multidisciplinary intervention team was established, led by nursing researchers who had received systematic rehabilitation training. The team consists of 1 nursing manager, 1 sports medicine doctor, 1 orthopedic joint team doctor, 2 charge nurses, 2 responsible nurses, 1 rehabilitation physician, and 3 nursing postgraduates. Researchers are responsible for patient enrollment, intervention implementation, data collection, and follow-up; supervisors provided overall project oversight; the director of the sports medicine department participates in guiding the research design; the orthopedic joint team doctors assist in formulating the rehabilitation plan; the charge nurses and responsible nurses supervised the intervention process; other members are respectively responsible for joint function assessment, questionnaire guidance, assistance in rehabilitation exercises, and assessment of rehabilitation training, etc. To ensure the uniformity and scientific of rehabilitation training, the professional rehabilitation physician in the team also conducted unified knowledge training and assessment for the 3 nursing postgraduates and responsible nurses in advance. The study includes a control group and an experimental group.

The control group receives routine nursing care, including admission education, distribution of manuals, exercise guidance, family supervision, and regular follow-up.

The experimental group received a HAPA-based rehabilitation exercise program in addition to routine clinical care. This intervention was developed by integrating the best available evidence on exercise interventions for patients undergoing TKA^23^ with HAPA theoretical framework that partitions health behavior change into two sequential phases. This theoretical framework divided health behavior changes into the motivational phase and the volitional phase. The motivational phase focused on behavioral intention formation, whereas the volitional phase emphasized on action initiation and maintenance.

Phase 1: Motivational phase (at admission).

Nurses conducted structured preoperative education to help patients recognize the positive benefits and potential risks of postoperative rehabilitation exercises as well as understand disease-related knowledge through systematic preoperative education, thereby stimulating their internal motivation and behavioral intentions to change their behavior intentions.

Phase 2 − 1: Volitional phase – Planning period (from preoperative to early postoperative period).

Based on established intentions, healthcare providers developed personalized rehabilitation plans and facilitate the transition from “wanting to exercise” to “knowing how to exercise”. This includes: (1) a phased exercise sequence—starting with warm-up, progressing to strength, and balance training; (2) safety protocols: stop exercising immediately and notify medical staff if experiencing intolerable knee pain, shortness of breath, or other severe discomforts; (3) individualized adjustments: reduce intensity/duration for physically weak or elderly patients.

Phase 2–2: Volitional phase – Execution and maintenance period (postoperative rehabilitation period).

Nurses assist patients in correctly performing rehabilitation movements through bedside demonstrations, guided practice and repeated training; and help patients overcome difficulties, establish confidence and behavioral patterns for long-term exercise, and ensure the maintenance and recovery of rehabilitation behaviors through continuous assessment of joint function.

**Fig. 1:**
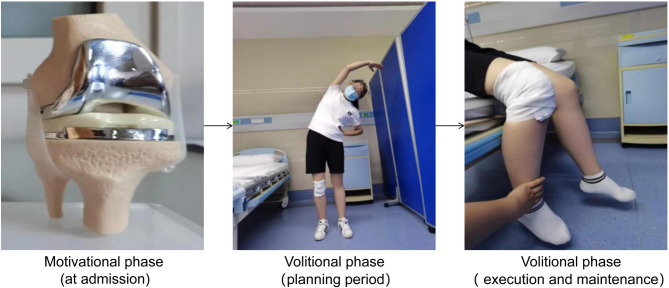
Representative photographs corresponding to the two phases (Motivational Phase, and two sub-stages of the Volitional Phase)of the HAPA-based postoperative rehabilitation program for TKA patients.

Finally, we integrated the HAPA with the best available evidence for exercise interventions in patients with TKA to develop a comprehensive postoperative rehabilitation program (see Supplementary 1). Figure 1 displays representative photographs corresponding to the two phases of this program-illustrating key practices across the motivational, planning, and execution/maintenance stages (see Supplementary 2 for the complete set).

### General information questionnaire

According to the research purpose and clinical conditions, the questionnaire was compiled after discussions among members of the research team. Its contents include patients’ basic information and clinical disease-related data.

### Hospital of special surgery (HSS)

Hospital of Special Surgery (HSS)^27^ has a total score of 100 points. The score sheet includes 7 items, among which 6 are scoring items: 30 points for pain, 22 points for functional activities (including walking, climbing stairs, and using public transportation), 18 points for joint range of motion, 10 points for muscle strength, 10 points for deformity, and 10 points for stability; the other item is a deduction item, which includes the use of walking sticks, limited extension, varus and valgus, etc. The results are divided into 4 grades: excellent (≥ 85 points), good (70–84 points), moderate (60–69 points), and poor (< 60 points).

### Western Ontario and McMaster Universities Osteoarthritis Index (WOMAC)

The Western Ontario and McMaster Universities Osteoarthritis Index (WOMAC) was proposed by Bellamy et al.^28^ in 1988. It is currently the most used assessment tool for evaluating knee osteoarthritis, consisting of 24 questions covering 3 aspects: the degree of joint pain (5 questions), the degree of joint stiffness (2 questions), and the degree of difficulty in daily life (17 questions). The levels of each degree are classified as none, mild, moderate, severe, and very severe, with corresponding scores of 0, 1, 2, 3, and 4 respectively. The total score is 96 points, and a higher score indicates a more severe condition.

### Self-efficacy for rehabilitation outcome scale (SER)

The Self-Efficacy for Rehabilitation Outcome Scale (SER) was developed by Professor Waldrop from the Miami Medical School in the United States in 2001 and is mainly used to evaluate the self-efficacy of patients undergoing hip and knee surgeries. In 2014, it was translated and introduced by Wang H Y et al.^29^. The Chinese version of SER includes two dimensions, namely self-efficacy for rehabilitation exercises (items 1–5) and self-efficacy for coping (items 6–12), with a total of 12 items. It adopts the Likert 11-level scoring method, ranging from 0 to 10, where 0 means “completely unable to do” and 10 means “no difficulty at all”. The total score is 120 points, and a higher score indicates a stronger sense of self-efficacy.

### Numerical rating scale (NRS)

Pain grading is evaluated according to the Numerical Rating Scale: 0 points indicate no pain, 10 points indicate unbearable severe pain, 1–3 points indicate mild pain, 4–6 points indicate moderate pain, and 7–10 points indicate severe pain.

### Range of motion (ROM)

Range of Motion (ROM) refers to the maximum angle each joint can reach from the neutral position (i.e., 0°) during joint movement. It is one of the basic indicators for assessing the scope and degree of damage to muscles, bones, neurological lesions, and joint movement functions. Assessment of knee joint range of motion^30^: A goniometer is used, with its axis located at the fibular head of the knee joint, the fixed arm parallel to the long axis of the femur, and the movable arm parallel to the long axis of the fibula to measure the degree of flexion and extension. Taking the neutral straight position as 0°, the angle when the lower leg touches the thigh during flexion is 140–150°, and the straight position is 0°. If hyperextension is possible, it is limited, approximately 5–10°.

### Muscle strength

The muscle strength was evaluated using the muscle strength item in the HSS scale, and it was classified into the following grades: complete ability to resist resistance (excellent, 10 points), partial ability to resist resistance (good, 8 points), ability to drive joint movement (moderate, 4 points), and inability to drive joint movement (poor, 0 points).

### Evaluation indicators

General data of the patients were collected during their hospitalization. Outcome indicators were measured at the following time points: preoperatively (T0), 1 day after surgery (T1), on the day of discharge (T2), 1 month after surgery (T3), and 3 months after surgery (T4). Primary outcome indicator was HSS. Secondary outcome indicators included the NRS, WOMAC, SER, ROM, and muscle strength.

### Quality control

In the research design stage, scientific rigor was ensured by selecting patients from the same ward, consulting experts, using authoritative tools, and adopting a non-concurrent controlled study. During the implementation stage, subjects were selected strictly in accordance with the inclusion and exclusion criteria to ensure the authenticity of the data. In the data collation and analysis stage, data entry was checked and verified by two people, and data analysis was conducted by other team members who did not participate in data collection to ensure the accuracy of the data.

### Statistical methods

Statistical analysis of the data was performed using SPSS 27.0 software. Enumeration data were expressed as frequencies and constituent ratios, and measurement data were expressed as means and standard deviations (x ± s). For data that met the conditions of normal distribution and homogeneous variance, the chi-square test was used for comparing enumeration data, and the t-test was applied for comparing measurement data. If the data did not meet the normal distribution, the non-parametric rank sum test was selected. Given the multiple comparisons involved in this study, False Discovery Rate (FDR) control was applied using the Benjamini-Hochberg procedure to maintain the false discovery rate below 5%. P-values reported for outcomes are those adjusted by the FDR method.

### Ethical principles

This study conformed to the Declaration of Helsinki and was approved by the Medical Ethics Committee of the First Affiliated Hospital of Kunming Medical University (approval number: 2022-L-62). Prior to the initiation of the study, the researchers provided a detailed explanation of the study purpose and procedures to all participants. After confirming their understanding and willingness to participate, all participants voluntarily signed the written informed consent form.

## Results

### Patient flow and basic characteristics

**Fig. 2:**
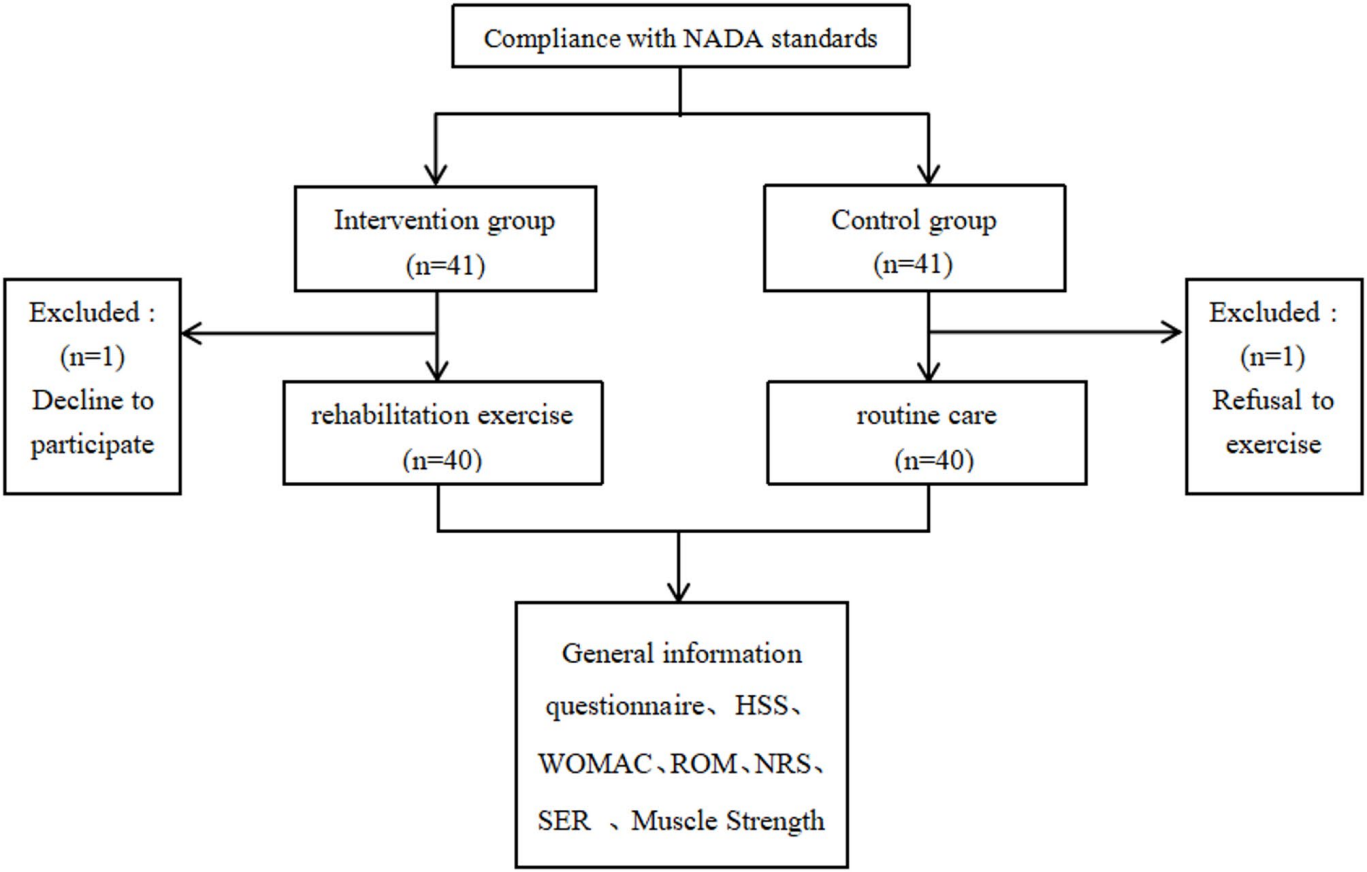
Participants’ flow.

Forty-one patients who underwent TKA were included in both the control group and the experimental group. During the research process, one patient in the control group had poor compliance after discharge, refused to cooperate with exercises and data collection, so he was excluded. One patient in the experimental group was unable to tolerate pain during hospitalization and was unwilling to continue participating in the study. Both the control group and the experimental group lost 1 case, and finally 40 patients were collected in each group (Fig. 2).

Among the two groups of patients who underwent knee replacement surgery, the proportion of female patients was relatively high (77.5% in the control group and 87.5% in the experimental group). The average age of the control group was (64 ± 12.92) years, and that of the experimental group was (65.75 ± 10.73) years. The educational level was mainly primary school or below (57.5% in the control group and 55% in the experimental group). The duration of illness in the control group was (6.14 ± 5.05) years, and that in the experimental group was (7.81 ± 6.82) years. The postoperative hospital stay in the control group was (9.53 ± 3.48) days, while that in the experimental group was (9.4 ± 4.05) days. Detailed demographic and clinical characteristics are shown in Table 1. In conclusion, the two groups were relatively balanced in terms of demographic and clinical characteristics.

**Table 1:** Demographic and clinical characteristics of the two groups of patients.

Project		Control group *n* = 40	Experimental group *n* = 40	Statistics (Z/x^2^)	*P*
Age (years)		64 ± 12.92	65.75 ± 10.73	− 0.659 ^b^	0.512
Sex	Male	9 (22.5)	5 (12.5)	1.385^a^	0.380
	Female	31 (77.5)	35 (87.5)		
Place of residence	Town	16 (40)	20 (50)	0.808^a^	0.500
	Rural	24 (60)	20 (50)		
Ethnic group	Han nationality	36 (90)	31 (77.5)	2.296^a^	0.130
	Ethnic minorities	4 (10)	9 (22.5)		
Marital status	Married	37 (92.5)	34 (85)	1.580^b^	0.710
	Divorced	1 (2.5)	1 (2.5)		
	Widowed	2 (5)	5 (12.5)		
Living situation	With spouse	19 (47.5)	13 (32.5)	4.860 ^b^	0.280
	With children	8 (20)	6 (15)		
	With spouses and children	11 (27.5)	19 (47.5)		
	Living alone	1 (2.5)	2 (5)		
	Others	1 (2.5)	0 (0)		
Educational level	Primary school and below	23 (57.5)	22 (55)	− 0.020 ^b^	0.980
	Junior high school	10 (22)	13 (32.5)		
	Senior high school	5 (12.5)	4 (10)		
	Junior college and above	2 (5)	1 (2.5)		
Occupation	Farmer	28 (70)	20 (50)	6.210^b^	0.130
	Worker	5 (12.5)	5 (12.5)		
	Self-employed person	0 (0)	1 (2.5)		
	Teacher	2 (5)	1 (2.5)		
	Others	5 (12.5)	13 (32.5)		
Source of income	Pension	14 (35)	11 (27.5)	0.560 ^b^	0.850
	Labor income	15 (37.5)	17 (42.5)		
	Provided by family members	11 (27.5)	12 (30)		
Type of medical insurance	At one’s own expense	0 (0)	2 (5)	3.340 ^b^	0.390
	Urban employee medical insurance	13 (32.5)	12 (30)		
	Urban and rural resident medical insurance	25 (62.5)	26 (65)		
	Others	2 (5)	0 (0)		
Affected limb	Left side	13 (32.5)	15 (37.5)	1.790 ^b^	0.520
	Right side	25 (62.5)	25 (62.5)		
	Both sides	2 (5)	0 (0)		
Preoperative self-care ability	Completely self-care	30 (75)	34 (85)	1.250^a^	0.260
	Partially dependent	10 (22)	6 (15)		
	Totally dependent	0 (0)	0 (0)		
Preoperative assistive devices	Yes	10 (22)	10 (22)	0.000^a^	1.000
	No	30 (75)	30 (75)		
Duration of illness (years)		6.14 ± 5.05	7.81 ± 6.82	− 1.244 ^b^	0.217
Postoperative hospital stays (days)		9.53 ± 3.48	9.4 ± 4.05	0.148 ^b^	0.883

a is the value of x^2^ and b is the value of Z.

### The effect of rehabilitation exercise intervention based on the HAPA theory on patients undergoing TKA

As shown in Table 2, no significant differences were found in the total HSS score, muscle strength, or other dimensions between the two groups of patients before surgery. The total HSS score of the experimental group was higher than that of the control group on the day of discharge, 1 month after surgery, and 3 months after surgery, with statistically significant differences (P-adjusted = 0.02). The pain at 3 months after surgery, functional score on the day of discharge, functional score at 1 month after surgery were all higher than those in the control group, with statistically significant differences (P-adjusted < 0.05).

**Table 2:** Comparison of main outcome indicators at different time points between the two groups.

Project	Test time	Control group	Experimental group	Statistics (Z)	*P*-adjusted
HSS total score	T0	44.38 ± 8.11	45.74 ± 7.29	− 0.785	0.561
	T1	31.07 ± 5.89	33.92 ± 8.18	− 1.788	0.195
	T2	50.79 ± 5.41	54.64 ± 5.71	− 3.095	0.020^*^
	T3	54.29 ± 4.67	57.74 ± 5	− 3.192	0.020^*^
	T4	56.31 ± 3.89	61.02 ± 6.67	− 3.858	0.020^*^
Pain	T0	6.88 ± 3.52	8.5 ± 3.24	− 2.034	0.129
	T1	5.63 ± 3.61	6.5 ± 3.43	− 1.094	0.443
	T2	9.13 ± 2.75	9.88 ± 3.1	− 1.106	0.443
	T3	10 ± 2.77	10.13 ± 2.88	− 0.200	0.885
	T4	10.5 ± 2.21	12.13 ± 2.5	− 2.897	0.020^*^
Function	T0	8.65 ± 1.23	8.68 ± 1.59	− 0.583	0.700
	T1	1.7 ± 1.07	1.5 ± 1.41	− 0.905	0.487
	T2	7.8 ± 1.62	8.68 ± 1.59	− 2.643	0.032^*^
	T3	8.1 ± 1.58	9.13 ± 1.18	− 3.141	0.020^*^
	T4	8.75 ± 0.98	9.43 ± 2.35	− 1.922	0.157
Stability	T0	8.63 ± 1.13	8.65 ± 0.95	− 0.075	0.964
	T1	9.95 ± 0.32	10 ± 0	− 1.000	0.446
	T2	8.7 ± 0.97	8.65 ± 0.95	− 0.235	0.880
	T3	9.05 ± 1.01	9.3 ± 0.97	− 1.128	0.443
	T4	9.2 ± 0.99	9.65 ± 3.03	− 0.397	0.813
Deformity	T0	5.68 ± 4.18	4.28 ± 3.8	− 1.861	0.168
	T1	9.95 ± 0.32	10 ± 0	− 1.000	0.446
	T2	8.4 ± 1.72	8.38 ± 1.6	− 0.237	0.880
	T3	8.68 ± 1.44	8.8 ± 1.32	− 0.319	0.857
	T4	8.98 ± 1.19	9.15 ± 1	− 0.539	0.715
Range of motion	T0	10.76 ± 3.04	10.31 ± 2.91	− 1.122	0.443
	T1	3.24 ± 2.52	4.82 ± 3.08	− 2.440	0.050
	T2	13.22 ± 1.04	13.62 ± 0.98	− 1.581	0.246
	T3	13.46 ± 0.83	13.89 ± 0.89	− 2.451	0.050
	T4	13.68 ± 0.93	13.94 ± 0.84	− 0.987	0.446
Muscle strength	T0	8.25 ± 1.88	8.85 ± 1	− 1.062	0.443
	T1	3.6 ± 1.98	4.1 ± 2.12	− 1.080	0.443
	T2	7.7 ± 2.05	8.5 ± 1.41	− 1.677	0.221
	T3	8.75 ± 1.48	9.15 ± 1	− 1.068	0.443
	T4	8.95 ± 1.01	9.3 ± 0.97	− 1.568	0.246
Deduction of points	T0	4.45 ± 1.92	3.53 ± 2.26	− 2.677	0.031*
	T1	3 ± 0	3 ± 0	0.000	1.000
	T2	4.15 ± 2.01	3.05 ± 2.32	− 2.910	0.020*
	T3	3.75 ± 1.69	2.65 ± 1.51	− 2.922	0.020*
	T4	3.75 ± 1.69	2.58 ± 1.47	− 3.152	0.020*

T0, T1, T2, T3 and T4 were preoperative, 1 day after surgery, discharge day, 1 month after surgery and 3 months after surgery; P-adjusted: means presented to control for the increased risk of Type I error due to multiple comparisons; * means P-adjusted < 0.05.

There were no statistically significant differences in any secondary outcome indicators between the two groups of patients before surgery (Tables 2 and 3). On the day of discharge, the total WOMAC score and pain dimension score of the experimental group were lower than those of the control group (P-adjusted < 0.05). At 3 months after surgery, the NRS score, total WOMAC score, as well as the pain and stiffness dimensions within WOMAC in the experimental group were all lower than those in the control group (P-adjusted < 0.05). The ROM of the experimental group was better than that of the control group at 1 day and 1 month after surgery (P-adjusted < 0.05). In addition, a comparison of postoperative rehabilitation self-efficacy between the two groups showed that the score of postoperative rehabilitation self-efficacy in the experimental group was higher than that in the control group, with a statistically significant difference (P-adjusted < 0.05). See Table 3 for details.

**Table 3:** Comparison of secondary outcome indicators at different time points between the two groups.

Project	Test time	Control group	Experimental group	Statistics (Z)	*P*-adjusted
NRS total score	T0	6.73 ± 1.96	6.8 ± 1.67	− 0.02	0.984
	T1	7.95 ± 1.2	7.38 ± 1.67	− 1.331	0.301
	T2	4.88 ± 1.56	4.55 ± 1.52	− 1.065	0.388
	T3	4.88 ± 1.56	4.55 ± 1.52	− 1.065	0.388
	T4	4.88 ± 1.56	3.83 ± 1.39	− 3.156	0.009*
ROM total score	T0	86.08 ± 24.29	82.5 ± 23.26	− 1.122	0.388
	T1	25.95 ± 20.18	38.55 ± 24.62	− 2.440	0.038*
	T2	105.38 ± 8.27	108.5 ± 7.94	− 1.581	0.219
	T3	106.88 ± 6.76	110.6 ± 7.43	− 2.450	0.038*
	T4	109 ± 7.61	111 ± 7.09	− 0.987	0.413
WOMAC total score	T0	46 ± 6.34	43.53 ± 6.64	1.704	0.192
	T2	40.65 ± 5.72	36.83 ± 6.04	2.907	0.016*
	T4	34.73 ± 6.62	30.53 ± 6.23	2.922	0.016*
Degree of pain	T0	10.1 ± 1.88	8.93 ± 3.03	− 1.886	0.136
	T2	7.8 ± 2.13	5.5 ± 2.41	− 4.012	0.006*
	T4	6.83 ± 1.95	4.23 ± 1.54	− 5.413	0.006*
Degree of stiffness	T0	4.23 ± 1.59	4.08 ± 1.14	− 0.488	0.719
	T2	3.9 ± 1.36	3.88 ± 1.11	− 0.178	0.897
	T4	3 ± 1.49	2 ± 1.06	− 3.425	0.006*
Degree of difficulty in life	T0	31.68 ± 4.68	30.53 ± 4.98	− 0.942	0.419
	T2	28.95 ± 4.34	27.45 ± 4.76	− 1.534	0.221
	T4	24.9 ± 5.08	24.3 ± 5.44	− 0.396	0.758
SER total score	T1	85.18 ± 12.55	94.98 ± 8.79	− 4.045	0.006*

T0, T1, T2, T3 and T4 were preoperative, 1 day after surgery, discharge day, 1 month after surgery and 3 months after surgery, * means P-adjusted < 0.05.

As shown in Table 4, there were statistically significant differences in the total HSS score, total NRS score, total ROM score, and total WOMAC score between the two groups of patients in terms of time effect and inter-group effect (*P* < 0.05), but no statistically significant difference was found in the time-by-group interaction effect (*P* > 0.05).

**Table 4:** Analysis of variance of repeated measurements of outcome indicators at different time points in two groups of patients.

Project	Time	F/Wald x^2^ time	*P*-adjust	F/Wald x^2^ Between groups	*P*-adjust	F/Wald x^2^ Interaction	*P*-adjusted
HSS total score	T0-T4	441.59	0.001*	9.237	0.008*	1.563	0.383
Pain	T0-T4	66.044	0.001*	7.766	0.010*	6.911	0.383
Function	T0-T4	14.711	0.005*	16.54	0.004*	6.641	0.383
Stability	T0-T4	184.697	0.001*	4.971	0.030*	4.596	0.463
Deformity	T0-T4	1.083	0.298	1.354	0.245	3.070	0.695
Range of motion	T0-T4	1004.714	0.001*	5.233	0.028*	5.748	0.383
Muscle strength	T0-T4	27.471	0.001*	15.191	0.004*	1.369	0.915
Deduction of points	T0-T4	15.621	0.005*	30.112	0.004*	0.297	0.990
NRS total score	T0-T4	311.683	0.001*	8.047	0.010*	5.467	0.383
ROM total score	T0-T4	984.386	0.001*	5.275	0.028*	5.888	0.383
WOMAC total score	T0-T4	217.334	0.001*	7.489	0.014*	1.430	0.383
Degree of pain	T0-T4	138.408	0.001*	51.819	<0.001*	4.485	0.383
Degree of stiffness	T0-T4	75.293	0.001*	5.526	0.028*	6.972	0.383
Degree of difficulty in life	T0-T4	67.941	0.001*	3.020	0.088	0.344	0.915

* means P-adjusted < 0.05.

## Discussion

This study investigates the impact of a rehabilitation exercise program grounded in the HAPA on patients undergoing knee replacement surgery. The findings indicate that the HAPA-based rehabilitation exercise program is more effective than conventional nursing approaches in alleviating pain and improving knee joint function. The rehabilitation outcomes demonstrate significant clinical value and relevance.

### Rehabilitation exercise provides sustained analgesic benefits beyond early postoperative recovery

Our findings indicate that the principal advantage of structured rehabilitation in pain management may not appear immediately; instead, it contributes to a crucial role in ensuring sustained and progressive pain relief. Although early postoperative pain (T1) was similar between the groups, the experimental group exhibited superior pain control at later stages (T4). These results are consistent with the conclusions drawn by Cai L. B. et al.^31^. This temporal pattern suggests that rehabilitation exercises do not simply address transient, surgically induced pain but may fundamentally alter the trajectory of chronic pain. The significant reductions in the WOMAC “Degree of pain” and NRS scores at the final follow-up further support this hypothesis. Importantly, the statistically significant interaction effect in pain scores between the two groups provides a deeper insight: rehabilitation not only alleviating pain but also modifies its recovery pattern, facilitating a faster and more sustained improvement.

The collective results suggest that early postoperative exercise guidance, including mobilization starting on the first day after surgery, significantly alleviates activity-related discomfort and pain. The consistency of these findings with various Enhanced Recovery After Surgery (ERAS) protocols^32–35^ further supports the incorporation of structured rehabilitation as an essential element of accelerated recovery pathways.

### Rehabilitation exercises enhance muscle strength and promote the recovery of joint function in for TKA patients

Following surgery, both groups demonstrated gradual improvements in function and muscle strength measured by the HSS score. However, the experimental group achieved higher scores than the control group. This outcome suggests that providing guidance and supervision in rehabilitation exercises post-surgery can significantly enhance muscle strength and joint function, corroborating the findings of Wei G^36^. It is noteworthy that both groups experienced a significant decline in HSS scores on the first postoperative day, followed by an upward trend in subsequent periods, while NRS scores increased. This initial decline in HSS scores must be attributed to the incomplete recovery of muscle strength and apprehension about movement caused by wound pain. From the first postoperative day to discharge, both groups showed significant increases in HSS scores. This aligns with previous studies^37–39^, which suggest that implementing rehabilitation exercises during hospitalization can enhance joint function.

Research conducted by Luo D^40^ demonstrated the high feasibility of performing follow-up assessments at 1 month, 3 months, 6 months, and beyond. Building on these findings, we conducted telephone follow-ups at 1 month and 3 months post-surgery to evaluate joint mobility and rehabilitation progress. While also reminding patients of scheduled follow-ups to enhance adherence to functional exercises. Proper timing and gradual progression of exercise intensity are imperative throughout the rehabilitation. According to the FITT-VP exercise prescription principles established by the American College of Sports Medicine (ACSM)^41^, individuals with limited physical fitness commence muscle strength training at an intensity of 40%-50% of their one-repetition maximum (1-RM) and participate in aerobic exercise at a frequency of at least three days per week. Our rehabilitation program recommended slightly higher intensity, 50–60% 1-RM and a minimum of five days per week of aerobic exercise^42^. This discrepancy may reflect specific characteristics of our study population. Future high-quality studies are needed to develop locally tailored intervention strategies in China.

### Rehabilitation exercises improve self-efficacy and quality of life in TKA patients

Through the implementation HAPA-guided rehabilitation training program, the experimental group demonstrated significantly lower WOMAC scores, and pain levels compared with the control group at discharge and at three months post-surgery. Furthermore, stiffness scores in the experimental group were markedly reduced at the three-month postoperative interval relative to the control group. These findings imply that early rehabilitation exercise guidance provided by a multidisciplinary team can effectively promote functional recovery and improve patients’ quality of life, supporting the conclusions drawn by Dutta S^43^. Moreover, rehabilitation self-efficacy scores were highly significant in the experimental group at three months after surgery, consistent with Zhang Y Y^44^, who reported that rehabilitation exercise guidance enhances self-efficacy among TKA patients. Additional studies^31,45,46^ corroborate these findings, demonstrating that multidisciplinary approaches increase patients’ confidence in the rehabilitation process.

In clinical practice, a multidisciplinary approach is essential for providing comprehensive rehabilitation exercise guidance. Healthcare professionals trained in rehabilitation techniques should lead functional exercises instructions. Prior to guiding patients, team members should receive standardized, evidence-based rehabilitation training to ensure adequate knowledge and skills to deliver safe and effective rehabilitation.

### Limitations

This study has several limitations. First, the non-concurrent quasi-experimental design, while necessitated by pandemic-related suspension of elective surgeries, introduces potential temporal bias despite statistical equivalence in baseline characteristics. Standardized completely randomized controlled trials should be conducted in the future to reduce selection bias and ensure balanced study groups. Second, outcome assessors were members of the intervention team and were not blinded, introducing potential detection bias; however, standardized assessment protocols and patient-reported outcomes (WOMAC) were employed to mitigate this concern. Third, the exclusion of dropouts without intention-to-treat analysis may have inflated effect sizes, though sensitivity analyses (Supplementary 3) using both last-observation-carried-forward and worst-case scenario methods demonstrated the robustness of our primary findings. Finally, the study deviated from CONSORT guidelines by lack of prospective trial registration, but retrospective registration has been completed with the Chinese Clinical Trial Registry (ChiCTR; registration No. [294441]) and relevant information is publicly accessible.

## Conclusions

In conclusion, the rehabilitation exercise intervention for TKA patients, developed by integrating the best evidence-based exercise interventions for TKA patients with HAPA theory, effectively reduced pain, improved joint function, increased muscle strength, and enhanced rehabilitation self-efficacy. Healthcare professionals are encouraged to incorporate this rehabilitation exercise program into the rehabilitation care plans and clinical practice and to tailor individualized, safe, and scientifically grounded exercise plans based on patient characteristics. This approach is expected to enhance early postoperative recovery efficiency, mitigate joint dysfunction, accelerate restoration of knee joint function, and improve patients’ overall quality of life.

## Supplementary Information

Below is the link to the electronic supplementary material.

Supplementary Material 1

## Data Availability

The data used in this research is available. Please send request to Prof. Ying Tian.

## References

[1] Driban, J. B. et al. Association of knee injuries with accelerated knee osteoarthritis progression: data from the osteoarthritis initiative. *Arthritis Care Res. (Hoboken)*. **66**, 1673–1679. 10.1002/acr.22359 (2014). 24782446 10.1002/acr.22359 PMC4211979

[2] Sanchez, C. A., Jara, A. B. & Marino, J. Superficial femoral artery pseudoaneurysm, compartment syndrome, and deep vein thrombosis after total knee arthroplasty. *Arthroplast Today*. **6**, 227–230. 10.1016/j.artd.2020.02.003 (2020). 32577468 10.1016/j.artd.2020.02.003 PMC7303499

[3] Cui, A. et al. Global, regional prevalence, incidence and risk factors of knee osteoarthritis in population-based studies. *EClinicalMedicine*. **29** (2020). 10.1016/j.eclinm.2020.100587 PMC7704420 34505846

[4] Zhu, S., Qu, W. & He, C. Evaluation and management of knee osteoarthritis. *J. Evid. Based Med.* **17**, 675–687. 10.1111/jebm.12627 (2024). 38963824 10.1111/jebm.12627

[5] Ackerman, I. N. et al. The projected burden of primary total knee and hip replacement for osteoarthritis in Australia to the year 2030. *BMC Musculoskelet. Disord*. **20**, 90. 10.1186/s12891-019-2411-9 (2019). 30797228 10.1186/s12891-019-2411-9 PMC6387488

[6] Mu, H. & Li, W. L. L. Design and application of knee rehabilitation trainer. *Chin. J. Nurs.* **54**(07), 1113–1115 (2019).

[7] Wirries, N., Ezechieli, M., Stimpel, K. & Skutek, M. Impact of continuous passive motion on rehabilitation following total knee arthroplasty. *Physiother Res. Int.* **25**, e1869. 10.1002/pri.1869 (2020). 32985036 10.1002/pri.1869

[8] Singh, J. A. Epidemiology of knee and hip arthroplasty: a systematic review. *Open. Orthop. J.* **5**, 80–85. 10.2174/1874325001105010080 (2011). 21584277 10.2174/1874325001105010080 PMC3092498

[9] Beard, D. J. et al. The clinical and cost-effectiveness of total versus partial knee replacement in patients with medial compartment osteoarthritis (TOPKAT): 5-year outcomes of a randomised controlled trial. *Lancet* **394**, 746–756. 10.1016/S0140-6736(19)31281-4 (2019). 31326135 10.1016/S0140-6736(19)31281-4 PMC6727069

[10] Neuprez, A. et al. Patients’ expectations impact their satisfaction following total hip or knee arthroplasty. *PLoS One*. **11**, e0167911. 10.1371/journal.pone.0167911 (2016). 27977711 10.1371/journal.pone.0167911 PMC5158008

[11] Peter, W. F., Nelissen, R. G. & Vlieland, T. P. Guideline recommendations for post-acute postoperative physiotherapy in total hip and knee arthroplasty: are they used in daily clinical practice? *Musculoskelet. Care*. **12**, 125–131. 10.1002/msc.1067 (2014). 10.1002/msc.1067 24497426

[12] Bartholdy, C. et al. The role of muscle strengthening in exercise therapy for knee osteoarthritis: A systematic review and meta-regression analysis of randomized trials. *Semin Arthritis Rheum.* **47**, 9–21. 10.1016/j.semarthrit.2017.03.007 (2017). 28438380 10.1016/j.semarthrit.2017.03.007

[13] Fransen, M. et al. Exercise for osteoarthritis of the knee: a cochrane systematic review. *Br. J. Sports Med.* **49**, 1554–1557. 10.1136/bjsports-2015-095424 (2015). 26405113 10.1136/bjsports-2015-095424

[14] Schiphof, D., van den Driest, J. J. & Runhaar, J. Osteoarthritis year in review 2017: rehabilitation and outcomes. *Osteoarthr. Cartil.* **26**, 326–340. 10.1016/j.joca.2018.01.006 (2018). 10.1016/j.joca.2018.01.006 29330103

[15] Michie, S. ABC of behaviour change theories. (2014).

[16] Clark, H. & Bassett, S. An application of the health action process approach to physiotherapy rehabilitation adherence. *Physiother Theory Pract.* **30**, 527–533. 10.3109/09593985.2014.912710 (2014). 24779488 10.3109/09593985.2014.912710

[17] Zhang, C. Q., Zhang, R., Schwarzer, R. & Hagger, M. S. A meta-analysis of the health action process approach. *Health Psychol.* **38**, 623–637. 10.1037/hea0000728 (2019). 30973747 10.1037/hea0000728

[18] Schwarzer, R., Lippke, S. & Luszczynska, A. Mechanisms of health behavior change in persons with chronic illness or disability: the health action process approach (HAPA). *Rehabil Psychol.* **56**, 161–170. 10.1037/a0024509 (2011). 21767036 10.1037/a0024509

[19] Meng, X. & Yu, Y. Effect of rehabilitation nursing under the guidance of the health action process approach model on perioperative nursing effect of artificial hip arthroplasty: effect on promoting quality of life and postoperative rehabilitation. *Comput. Math. Methods Med.* **2022**, 1247002. 10.1155/2022/1247002 (2022). 10.1155/2022/1247002 PMC9019436 35465014

[20] Zhang, C. et al. Theoretical explanation of upper limb functional exercise and its maintenance in postoperative patients with breast cancer. *Front. Psychol.* **12**, 794777. 10.3389/fpsyg.2021.794777 (2021). 35069382 10.3389/fpsyg.2021.794777 PMC8766984

[21] Duan, Y. et al. A WeChat mini program-based intervention for physical activity, fruit and vegetable consumption among Chinese cardiovascular patients in Home-Based rehabilitation: A study protocol. *Front. Public. Health*. **10**, 739100. 10.3389/fpubh.2022.739100 (2022). 35392478 10.3389/fpubh.2022.739100 PMC8980353

[22] Pozzi, F., Snyder-Mackler, L. & Zeni, J. Physical exercise after knee arthroplasty: a systematic review of controlled trials. *Eur. J. Phys. Rehabil Med.* **49**, 877–892 (2013). 24172642 PMC4131551

[23] Huang, Y. & Li Yl, Y. Q. Evidence summary of exercise intervention in patients undergoing knee arthroplasty. *Chin. J. Nurs.* **58**(18), 2265–2272 (2023).

[24] Wang, K. Osteoarthritis treatment guidelines. *Chin. J. Orthopaed*. **6**, 705–715 (2018).

[25] Liang, X. Y. et al. Application of fast tract surgery in nursing for patients undergoing total knee arthroplasty. **19**(24), 28–30 (2012).

[26] Wei, H. H. Construction and empirical study of rehabilitation nursing scheme for total knee arthroplasty underenhanced recovery after surgery. *Xinjiang Med. University* (2020).

[27] Insall, J. N., Ranawat, C. S., Aglietti, P. & Shine, J. A comparison of four models of total knee-replacement prostheses. *J. Bone Joint Surg. Am.* **58**, 754–765 (1976). 956219

[28] McConnell, S., Kolopack, P. & Davis, A. M. The Western Ontario and McMaster Universities Osteoarthritis Index (WOMAC): a review of its utility and measurement properties. *Arthritis Rheum.* **45**, 453–461. 10.1002/1529-0131(200110)45:5%3C453::aid-art365%3E3.0.co;2-w (2001). 11642645 10.1002/1529-0131(200110)45:5<453::aid-art365>3.0.co;2-w

[29] Wang, H. Y. & Hu, X. Y. L. Evaluation of the reliability and validity of Chinese version self-efficacy for rehabilitation outcome scale. *Chin. J. Mod. Nurs.* **20**(3), 268–270 (2014).

[30] Xu, Y. H. & Wan, W. J. H. Observation of effect of warming needle moxibustion on Yanglingquan point and isokinetic training on stiff knee. *Mod. J. Integr. Chin. Western Med.* **24**(01), 15–17 (2015).

[31] Cai, L. B. et al. Does a program based on cognitive behavioral therapy affect kinesiophobia in patients following total knee arthroplasty? A randomized, controlled trial with a 6-month follow-up. *J. Arthroplasty*. **33**, 704–710. 10.1016/j.arth.2017.10.035 (2018). 29239772 10.1016/j.arth.2017.10.035

[32] Liu, H. W. Enhanced recovery after surgery in older patients with total knee arthroplasty: A systematic review and meta-analysis. *J. Orthop.* **68**, 230–237. 10.1016/j.jor.2025.05.060 (2025). 40709050 10.1016/j.jor.2025.05.060 PMC12284530

[33] Salamanna, F. et al. Key Components, current practice and clinical outcomes of ERAS programs in patients undergoing orthopedic surgery: A systematic review. *J. Clin. Med.* **11**10.3390/jcm11144222 (2022). 10.3390/jcm11144222 PMC9322698 35887986

[34] Hardy, A. et al. Comparing ERAS-outpatient versus standard-inpatient hip and knee replacements: a mixed methods study exploring the experience of patients who underwent both. *BMC Musculoskelet. Disord*. **22**, 978. 10.1186/s12891-021-04847-9 (2021). 34814889 10.1186/s12891-021-04847-9 PMC8611950

[35] Morrell, A. T. et al. Enhanced recovery after primary total hip and knee arthroplasty: A systematic review. *J. Bone Joint Surg. Am.* **103**, 1938–1947. 10.2106/JBJS.20.02169 (2021). 34166275 10.2106/JBJS.20.02169

[36] Wei, G., Shang, Z., Li, Y., Wu, Y. & Zhang, L. Effects of lower-limb active resistance exercise on mobility, physical function, knee strength and pain intensity in patients with total knee arthroplasty: a systematic review and meta-analysis. *BMC Musculoskelet. Disord*. **25**, 730. 10.1186/s12891-024-07845-9 (2024). 39267026 10.1186/s12891-024-07845-9 PMC11395693

[37] Lorbeer, N. et al. Volitional processes in changing physical activity: A randomized controlled trial with individuals with knee osteoarthritis. *Health Psychol.* **44**, 597–607. 10.1037/hea0001453 (2025). 39679978 10.1037/hea0001453

[38] Li, L. & Yin, W. Z. Effect of early gait training on the functional rehabilitation after total knee arthroplasty. *Chin. J. Tissue Eng. Res.* **21**, 4288–4293 (2017).

[39] Sattler, L. N., Hing, W. A. & Vertullo, C. J. Pedaling-based protocol superior to a 10-exercise, non-pedaling protocol for postoperative rehabilitation after total knee replacement: A randomized controlled trial. *J. Bone Joint Surg. Am.* **101**, 688–695. 10.2106/JBJS.18.00898 (2019). 30994586 10.2106/JBJS.18.00898

[40] Luo, D. Design and application of a home orthopaedic care platform. *Nurs. Res.* **32**(11), 1809–1811 (2018).

[41] Kluwer, W. *ACSM’s Guidelines for Exercise Testing and Prescription.*, Vol. 11th Edition. (American College of Sports Medicine, 2021). 10.1249/JSR.0b013e31829a68cf 23851406

[42] van Doormaal, M. C. M., Meerhoff, G. A., Vliet Vlieland, T. P. M. & Peter, W. F. A clinical practice guideline for physical therapy in patients with hip or knee osteoarthritis. *Musculoskelet. Care*. **18**, 575–595. 10.1002/msc.1492 (2020). 10.1002/msc.1492 32643252

[43] Dutta, S., Ambade, R., Wankhade, D. & Agrawal, P. Rehabilitation techniques before and after total knee arthroplasty for a better quality of life. *Cureus* **16**, e54877. 10.7759/cureus.54877 (2024). 38533163 10.7759/cureus.54877 PMC10965116

[44] Zhang, Y. Y. Construction and application of an IMB model-based rehabilitation exercise programme for total knee replacement patients. (Qingdao University, 2022).

[45] Cai, L. B. & Li, L. Y. J. A study of a collaborative multidisciplinary intervention programme in patients with agoraphobia after total knee arthroplasty. *Chin. J. Nurs.* **55**(04), 494–499 (2020).

[46] Kim, S., Hsu, F. C., Groban, L., Williamson, J. & Messier, S. A pilot study of aquatic prehabilitation in adults with knee osteoarthritis undergoing total knee arthroplasty-short term outcome. *BMC Musculoskelet. Disord.* **22**(1), 388 (2021). 33902505 10.1186/s12891-021-04253-1 PMC8074697

