# Supplementary material for: Effect of the health action process approach theory in patients undergoing total knee arthroplasty
Source: Sci Rep. 2026 Jan 5;16:4469. doi: 10.1038/s41598-025-34635-7 (PMC12864869; doi:10.1038/s41598-025-34635-7)
Supplement: Supplementary file 1 — Supplementary Material 1 [file 41598_2025_34635_MOESM1_ESM.pdf]

Supplementary 1. Rehabilitation exercise intervention measures for patients after TKA

HAPA stage	Intervention time	Intervention content	Exercise frequency and duration	Intervention methods	Intervening personnel
motivational stage	S0	(1) Conduct admission education for patients<br>(2) Establish a good relationship between nurses and patients<br>(3) Assess preoperative knee function	30min.	Video education, face-to-face, model display, rehabilitation exercise guidebook.	Researchers, attending physicians, responsible nurses
	S1	(1) Teach patients exercise before surgery:<br>① Ankle pump exercise; ② Straight leg raise training; ③ Joint mobility training: press the knee joint; ④ Muscle strength training; ⑤ Using a walking aid and other training<br>(2) Supervision exercise:<br>The family members assisted the patients to carry out preoperative rehabilitation exercise guidance, and supervised the rehabilitation exercise for patients with cognitive impairment, difficult to manage daily life, persistent functional damage, and unable to achieve self-rehabilitation.	Do 3 sets of each exercise, 10 times per set, with a gap of 30-60s between two sets, and the total exercise time is 30min.	Video education, face-to-face, model display, bedside practice, rehabilitation exercise guidebook.	Same as above.
volitional stage	S2 (before exercise)	Inform the pre-sport precautions:<br>(1) Consider the special needs and preferences of patients. For the physically weak and older patients, the intensity of exercise can be appropriately reduced and the time shortened; for the patients with good physical fitness and faster recovery, the intensity can be gradually increased during the training process and follow the training principles.<br>(2) Training principles: Start with warm-up exercises such as slow walking,	Warm up exercise time is 15min.	Same as above.	Same as above.

	marching on the spot or stretching muscles, transition to mobility, flexibility, strength and balance training, and end with relaxation exercises. Start with guided exercise therapy once or twice a week until you can exercise independently and gradually reduce guidance during treatment.<br>(3) If you have chest pain, chest tightness, shortness of breath, palpitations, dizziness and other discomfort during exercise, please stop exercising immediately and inform the medical staff in time.			
S2	(1) In the morning ward round or shift handover, instruct patients to do functional exercises in bed.<br>(2) Mobilize patients to use walking aids to get out of bed and require family members to participate in the whole process of exercise to ensure patient safety.<br>(3) Functional exercises: ① Ankle pump exercise; ② Straight leg raise training; ③ Joint mobility training: press the knee joint; ④ Muscle strength training, see Rehabilitation Exercise Guidebook (Part 4: Functional Exercises)<br>(4) Supervise patients' functional exercise<br>(5) Assess knee function	(1) Do 3 sets of each exercise, 10 times per set, with a gap of 30-60s between two sets, and the total exercise time is 30min.<br>(2) Supervision and exercise: supervise and exercise patients in the morning ward round, at the end of treatment in the morning, and at the afternoon shift handover.	Same as above.	Same as above.
S3	(1) Functional exercises: ① Ankle pump exercise; ② Straight leg raise training; ③ Joint mobility training: press the knee joint; ④ Muscle strength training; ⑤ use of walking aid; ⑥ Passive flexion and extension function exercises of knee joint	Same as above.	Same as above. guidebook	Researcher, responsible nurse
S4	(1) Functional exercises: ① Ankle pump exercise; ② Straight leg raise tr	Same as above.	Same as above.	Same as

	aining; ③ Joint mobility training: press the knee joint;④Muscle strength training; ⑤ use of walking aid; ⑥ Passive flexion and extension function exercises of knee joint			above.
S5	(1) Functional exercises: ① Ankle pump exercise; ② Straight leg raise training; ③ Joint mobility training: press the knee joint; ④ Muscle strength training; ⑤ use of walking aid; ⑥ Passive flexion and extension function exercises of knee joint	Do 3 sets of each exercise, 15 times per set, with a gap of 30-60s between two sets, and the total exercise time is 45min.	Same as above.	Same as above.
S6	(1) Functional exercises: ① Ankle pump exercise; ② Straight leg raise training; ③ Joint mobility training: press the knee joint; ④ Muscle strength training; ⑤ use of walking aid; ⑥ Passive flexion and extension function exercises of knee joint; ⑦ Active flexion and extension of the knee joint.	Do 4 sets of each exercise, 15 times per set, with a gap of 30-60s between two sets, and the total exercise time is 60min.	Same as above.	Same as above.
S7	(1) The rehabilitation exercise content is the same as S6<br>(2) At discharge, patients were informed to have outpatient review at 1 month, 3 months, 1 year and 2 years to check the incision condition and evaluate the joint function status. The contents are shown in Rehabilitation Exercise Guidebook (Part 6: Discharge Guidance)<br>(3) Assess knee function	Same as above.	Rehabilitation exercise guidebook.	Same as above.
S8	(1) Adhere to the rehabilitation exercise during hospitalization<br>(2) Supervision exercise:<br>① In the second week after discharge, remind patients to go to the outpatient clinic for a follow-up visit one month after surgery by phone or wechat, and at the same time, understand the situation of patients' rehabilitation		Rehabilitation exercise guidebook, wechat + telephone	Researchers, 2 graduate students

	exercise and the implementation of family cooperative care, ask them to r epeat the exercise movements and send them to members of the research group;<br>② Ask the patient about the range of motion and pain of the knee joint, urge them to check in every day in the wechat group, and send tweets a nd videos about rehabilitation exercises every morning. For the relevant qu estions of the patients, the researchers will reply in time;<br>③ For patients with poor joint activity and low social support, the freque ncy of urging should be increased, and personal reminders should be sent via wechat every week. For patients with poor compliance, video supervisi on should be carried out via wechat.		follow-up.	
S9	(1) Adhere to the rehabilitation exercise during hospitalization. On this basi s, aerobic exercise, muscle strength training, functional training, etc. can be carried out. See Rehabilitation Exercise Guidebook (Part 5: Other exercise s).<br>(2) Supervision and exercise;<br>① For patients who came to the hospital for follow-up one month after s urgery, they were reminded by phone at two months after surgery to have an outpatient review three months after surgery;<br>② For patients who will be re-examined in the outpatient department in t he future, they will be reminded every two weeks and urged by video at t he same time;<br>③ Other measures are the same as those during "S8".	During hospitalization, the intensity and tim e of exercise should b e increased on the ba sis of functional exerc ise	Rehabilitation exercise guidebook, wechat +telephone follow-up.	Researchers, 2 graduate students

S0: 1 week before the surgery

S1: 2-3 days before the surgery

S2: Day 1 after surgery

S3: Day 2 after surgery

S4: Day 3 after surgery

S5: Day 4 after surgery

S6: Day 5 after surgery

S7: From the 6th day after surgery to the day of discharge

S8: From the day of discharge to 1 month after surgery

S9 :From 1 month to 3 months after surgery

Supplementary 2

Rehabilitation Exercise Guidebook for Patients Undergoing Knee Arthroplasty Surgery

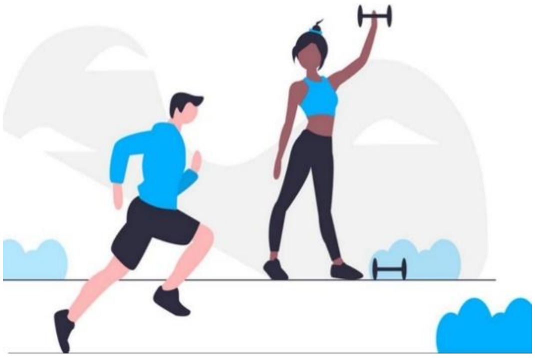

Table of Contents

1.Why Knee Arthroplasty is Performed··································1-2

2.The Benefits of Exercise··3

3.Precautions During Exercise··4-5

4.Functional Exercises···6

（1）Preoperative Functional Exercises································6-10

（2）Bedside Defecation Training···10

（3）Postoperative Muscle Strength Exercises ·····························11

（4）Postoperative Joint Range of Motion Exercises···················11-14

5.Other Exercises···15

（1）Aerobic Exercise···15

（2） Resistance Exercise··15

（3） Aquatic Therapy··16

（4）Balance Training··16

6.Discharge Instructions···17-19

1.Why Knee Arthroplasty is Performed?

1.Anatomical Site: The knee joint consists of the lower part of the thigh bone (femur), the kneecap (patella), and the top of the lower leg bones (taking the right knee as an example, Figure 1)

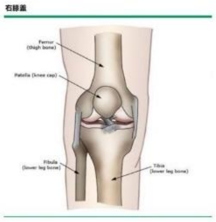

Figure 1

2. Knee Osteoarthritis: It refers to a knee joint disease caused by various factors that lead to knee joint cartilage fibrosis, cracking, ulceration, loss, and other conditions. When people have problems with their knee joints, the knees may experience pain, swelling, and stiffness, making it impossible to move normally.

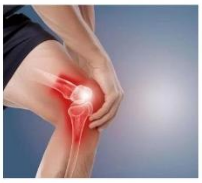

Pain

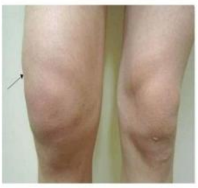

Swelling

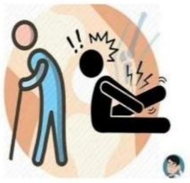

Stiffnes

3. Knee Arthroplasty: It refers to the removal of joint surfaces that the body can no longer repair on its own, and the replacement of the damaged joint with artificial jointcomponents.

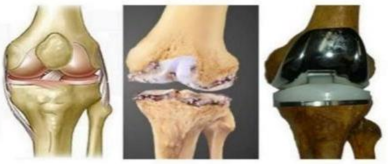

① ② ③

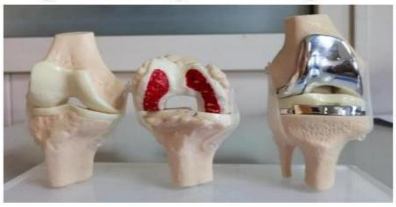

articular surface

①Normal ②damaged ③After Knee Arthroplasty

2.The Benefits of Exercise

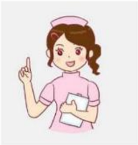

You can understand and be familiar with the help and benefits that rehabilitation exercises can bring to you.

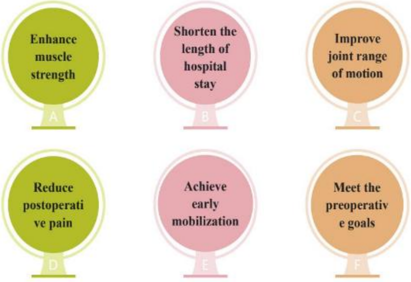

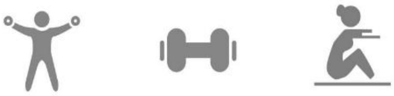

3.Precautions during exercise

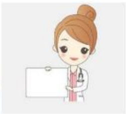

1. If you experience intolerable knee pain, shortness of breath, dizziness, fatigue, or other discomforts during exercise, you must stop exercising immediately and inform the medical staff. 2. When performing functional exercises, start with warm-up activities such as slow walking, marching in place, or muscle stretching. Then proceed with a certain amount of mobility, flexibility, strength, and balance training. Finally, end with relaxation exercises. 3. For special groups, such as those who are physically weak or elderly, the intensity and duration of exercise can be appropriately reduced. For patients with better physical fitness and faster recovery, the intensity should be gradually increased during training, while abiding by the training principles.

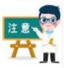

1. Stop exercising immediately when the following situations occur! !

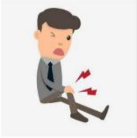

Intolerable Pain

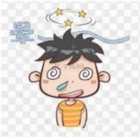

Dizziness

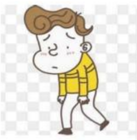

Fatigue

2. Start with warm-up exercises, then transition to strength training (a 500ml bottle of mineral water can be used as a substitute) and balance training.

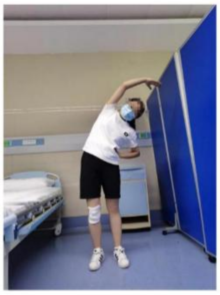

Warm-up exercises

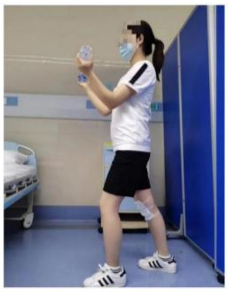

Strength training

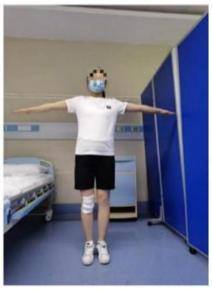

Balance training

4.Functional Exercises

(1).Preoperative Functional Exercises: You can start basic movement training before surgery and increase the number of repetitions according to your own conditions and practice every day until the day of the surgery.

(2)Functional Exercises: Ankle pump exercises, straight leg raise training, isometric contraction exercises of the quadriceps, cough training, bed defecation training, and walker use (mainly to help you adapt to functional exercises as early as possible after surgery; the duration and frequency of exercises can be adjusted according to your tolerance).

①Ankle pump exercise: Lie flat on the bed, extend your lower limbs, and relax your thighs. Slowly curl your toes inward, trying your best to point them toward yourself, hold for 3 seconds when reaching the maximum extent, then straighten and press your toes downward, hold for 3 seconds when reaching the maximum extent, and then relax. Do 20-50 times per set, and repeat 3 sets every day.

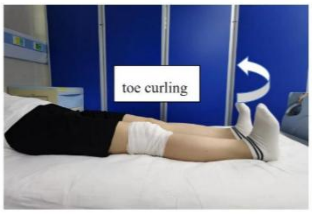

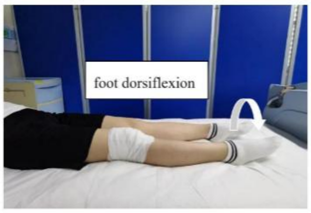

②Straight leg raise: Lift the affected limb until the lower leg forms a 30° angle with the horizontal plane, keep the knee joint straight, and dorsiflex the instep to tense the calf muscles. Hold the lifted position for a few seconds, then slowly lower it. Do 20-50 repetitions per set, and repeat 3 sets every day.

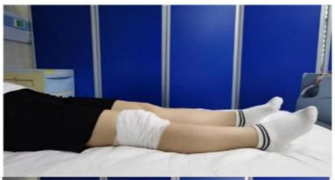

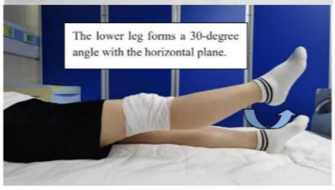

③Pressing the knee joint: Lie flat on the bed, straightens their legs naturally, then places a sandbag (table salt can be used as a substitute) on the you knee to keep the knee joint straight. Try to make the back of the knee closely against the bed. Hold this position for 5-10 seconds each time, repeat it several times, with 10-20 repetitions per set, and 3 sets per day.

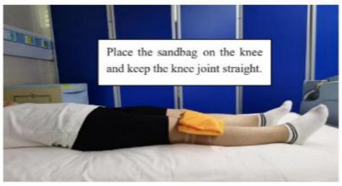

④Quadriceps isometric contraction exercise: Keep both legs straight, repeatedly tense and relax the muscles at the front of the thighs with force. Maintain the knee joint in a straight position for 5-10 seconds, then relax. Do 10-20 repetitions per set, and repeat 3 sets every day.

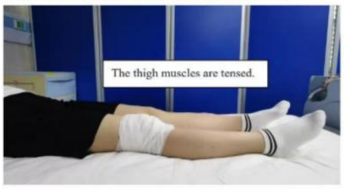

⑤Passive flexion and extension functional exercises of the knee joint:

Passive knee extension exercise: You should lie in a supine or prone position, with a pillow placed under the heel. Apply a downward force above the knee joint. When in a sitting position, hold the ankle with the hand to straighten the knee joint as much as possible and maintain this position for 10 seconds until a soreness is felt at the back of the knee. Do 10 times per set and repeat 3 sets.

Passive knee flexion exercise: You shouldt lie in a supine position or sits, with an auxiliary backward force applied to the front of the lower leg to flex the knee joint to the target angle. Maintain this position for 10 seconds, then straighten the knee joint again. Do 10 times per set and repeat 3 sets every day.

Passive knee extension in the supine position

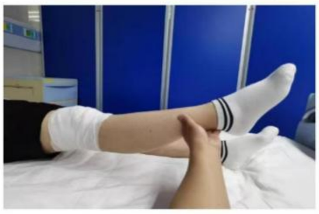

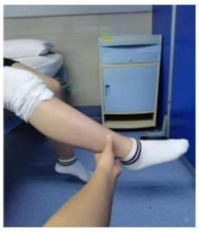

Passive knee extension in sitting position

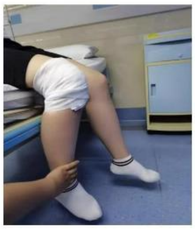

Passive knee flexion in sitting position

⑥Active flexion and extension exercise for the knee joint: Relying on the your own strength, actively perform knee joint flexion and extension training, with 10 repetitions per set and 3 sets repeated every day.

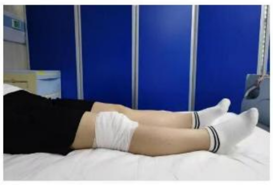

Active knee extension in sitting position

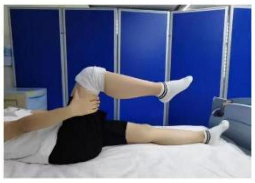

Active knee flexion in sitting position

⑦Breathing training (to enhance lung function and prevent lung infections): Inhale through the nose and exhale through the mouth.

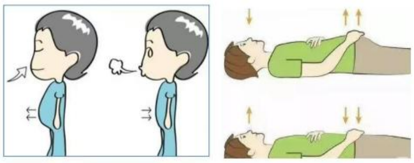

(2) Bedside Defecation Training

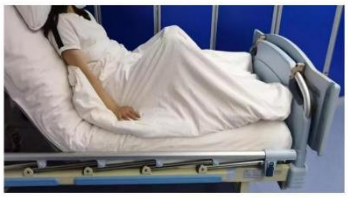

Postoperative exercise:

You can mobilize to get out of bed and move around using a walker on the first day after surgery, and walk in the ward and the department's corridor.

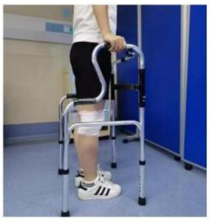

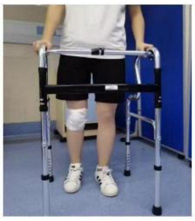

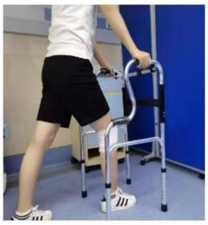

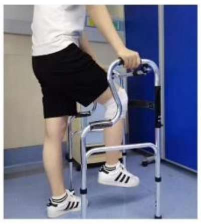

(3)Postoperative Muscle Strength Exercises(Same as before surgery): You can perform exercises in bed, starting with ankle pump exercises, straight leg raising exercises, knee compression, isometric contraction exercises of the quadriceps, etc, and then transitioning to passive flexion and extension exercises of the knee joint, active flexion and extension exercises of the knee joint, and so on.

(4)Postoperative Joint Range of Motion Exercises: Short and frequent sessions, avoiding joint swelling and pain.

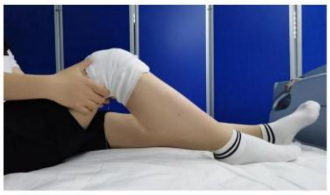

Flexing the knees while sitting or lying down

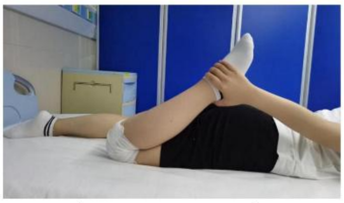

Flexing the knees while lying prone

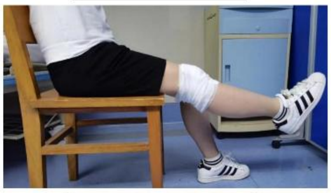

Extending the knee in a sitting position

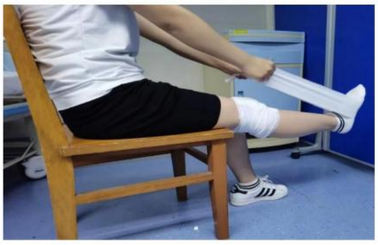

Hold the bandage and lift the lower leg upward.

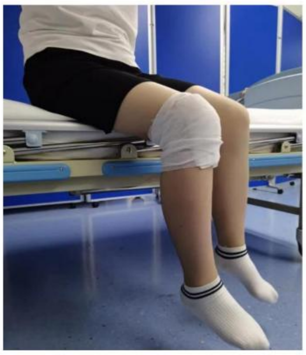

Actively flex the knee with the lower leg hanging over the edge of the bed.

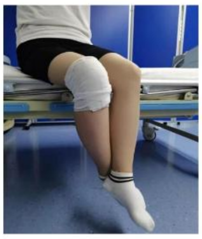

The unaffected limb presses down on the affected limb.

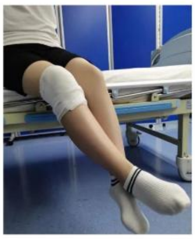

The unaffected limb lifts the affected limb upward.

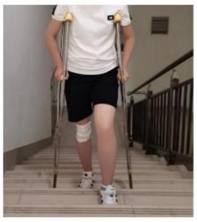

Going upstairs: first the unaffected limb, then the affected limb.

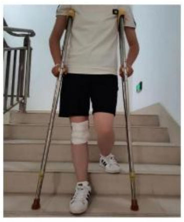

Going downstairs: first the affected limb, then the unaffected limb.

5.Other exercises

(1)Aerobic exercise: Aerobic training can be performed after postoperative functional recovery (for 6 months). Exercise frequency: at least 5 days a week, 30 minutes each time; Exercise intensity: the minimum intensity is ≥60% HRR (heart rate reserve); For those with poor physical fitness, they can transition from moderate intensity (40%-60% HRR) to higher intensity. It is necessary to ensure that the intensity is gradually increased during training and follow the training principles, such as brisk walking, cycling, swimming, and jogging.

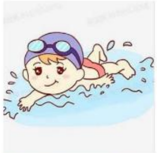
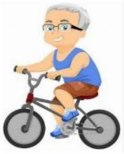
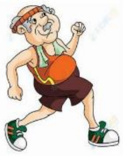

(2)Resistance exercise: Progressive resistance training should be carried out after surgery to improve walking speed and enhance lower limb muscle strength.

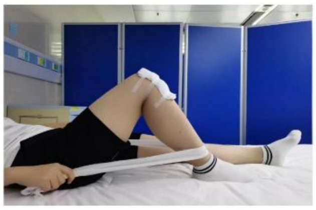

(3)Aquatic therapy: Aquatic exercises can be performed after surgery. The water temperature is generally 35-38℃, and exercising in warm water can reduce the pressure on the joints.

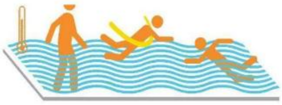

(4)Balance training: Conduct balance training after surgery, such as single-leg standing test, sit-to-stand test, balance board games, etc., to promote postoperative functional recovery.

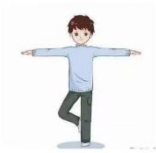
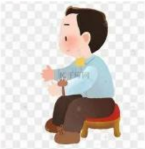
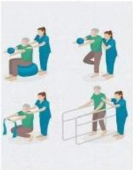

6.Discharge Instructions

You can be discharged when they achieve 0-5° of knee extension and 110-120° of knee flexion on the 5th to 7th day after surgery. After discharge, they should adhere to functional exercises as per the rehabilitation exercise guidebook, conduct regular re-examinations, and return to the hospital promptly if any abnormalities occur. Meanwhile, they should strengthen aerobic exercises, muscle strength training, and functional training, ensuring that the intensity is gradually increased during training and that training principles are followed.

Precautions:

◆When lying in bed, place a pillow under the heel of the operated limb to keep the knee joint extended and prevent flexion contracture.

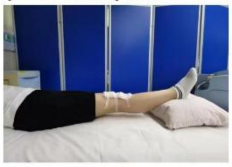

◆High-impact sports such as football and running are not recommended.

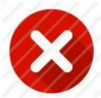
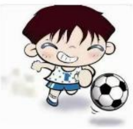
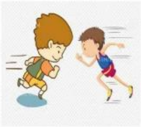

◆Low to moderate intensity exercises such as walking, cycling, and swimming can be performed.

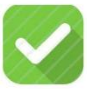
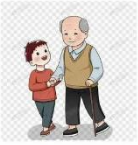
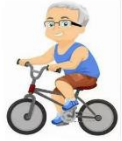
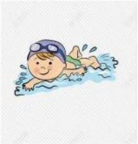

◆Fall Prevention: After surgery, move with the help of family members, medical and nursing staff using crutches or a walker. Partial weight-bearing walking with the affected limb can be performed 3 months after surgery, and the activity should be adjusted according to one's ability. If there is any discomfort, please go to the outpatient clinic for re-examination. Gradually increase the weight-bearing and walking distance according to the suggestions of the outpatient doctor. After surgery, prevent falls to avoid fractures, joint dislocations, etc.

♦Regular follow-up visits should be conducted after surgery to check the condition of the incision, guide patients in rehabilitation exercises, and evaluate joint function. The follow-up schedule is at 6 weeks, 3 months, 6 months, 1 year, and 2 years.

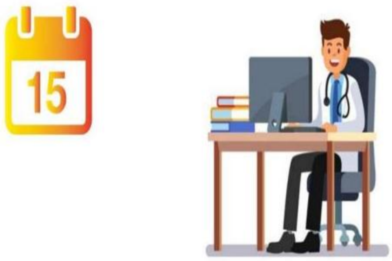

Supplementary 3. Sensitivity Analysis of HSS Total Score at T4

Analysis Method	Experimental group	Control group	Between-Group Difference	P-value
Original (Per-Protocol)	61.02±6.67	56.31±3.89	4.71	＜0.001
LOCF	60.52±7.32	56.07±4.13	4.45	0.007
Worst-Case Scenario	61.02±6.58	56.31±3.84	4.71	0.007

Abbreviation: LOCF, Last Observation Carried Forward.

(1)Last Observation Carried Forward (LOCF): This method assumes that dropouts showed no further improvement or deterioration after their last assessment. As shown in the table, the between-group difference remained statistically significant (P = 0.007) under this assumption, with only a minimal attenuation of the effect size (from 4.71 to 4.45).

(2)Worst-Case Scenario Analysis: In this highly conservative approach, we assumed that all dropouts in the experimental group had the worst-recorded outcome from their group at T4, while all dropouts in the control group had the best-recorded outcome. Reassuringly, even under this extreme and stringent assumption, the statistical significance of the intergroup difference was maintained (P = 0.007).
